# The lung-gut crosstalk in respiratory and inflammatory bowel disease

**DOI:** 10.3389/fcimb.2023.1218565

**Published:** 2023-08-23

**Authors:** Baoxiang Du, Yan Fu, Yuxiu Han, Qihui Sun, Jinke Xu, Yong Yang, Rong Rong

**Affiliations:** ^1^ College of Pharmacy, Shandong University of Traditional Chinese Medicine, Jinan, China; ^2^ Shandong Center for Disease Control and Prevention, Jinan, China; ^3^ Shandong Antiviral Engineering Research Center of Traditional Chinese Medicine, Shandong University of Traditional Chinese Medicine, Jinan, China; ^4^ Experimental Center, Shandong University of Traditional Chinese Medicine, Jinan, China; ^5^ Key Laboratory of Traditional Chinese Medicine Classical Theory, Ministry of Education, Shandong University of Traditional Chinese Medicine, Jinan, China; ^6^ Shandong Provincial Key Laboratory of Traditional Chinese Medicine for Basic Research, Shandong University of Traditional Chinese Medicine, Jinan, China

**Keywords:** lung-gut crosstalk, common mucosal immune system, respiratory disease, inflammatory bowel disease, microbiota

## Abstract

Both lung and gut belong to the common mucosal immune system (CMIS), with huge surface areas exposed to the external environment. They are the main defense organs against the invasion of pathogens and play a key role in innate and adaptive immunity. Recently, more and more evidence showed that stimulation of one organ can affect the other, as exemplified by intestinal complications during respiratory disease and *vice versa*, which is called lung-gut crosstalk. Intestinal microbiota plays an important role in respiratory and intestinal diseases. It is known that intestinal microbial imbalance is related to inflammatory bowel disease (IBD), this imbalance could impact the integrity of the intestinal epithelial barrier and leads to the persistence of inflammation, however, gut microbial disturbances have also been observed in respiratory diseases such as asthma, allergy, chronic obstructive pulmonary disease (COPD), and respiratory infection. It is not fully clarified how these disorders happened. In this review, we summarized the latest examples and possible mechanisms of lung-gut crosstalk in respiratory disease and IBD and discussed the strategy of shaping intestinal flora to treat respiratory diseases.

## 1 Introduction

Both the lung and gut are derived from the foregut region of the endoderm (Li, 2012). Although it is anatomically far away, when we stimulated one organ, the other compartment will also be affected, which indicates a vital communication between the gut and lung. In 1968, Warwick Turner first reported the existence of lung-gut crosstalk (Warwick turner, 1968), subsequently, with the development of novel technology and tools, various respiratory diseases including small or large airway dysfunction, lung parenchyma, and bronchitis have been found successively in patients with gastrointestinal diseases, especially in inflammatory bowel disease (IBD) (Rogers et al., 1971; Ceyhan et al., 2003; Larsen et al., 2010; Ramírez-Romero et al., 2017; Di Michele, 2022; Majima et al., 2022). Common respiratory diseases, such as asthma, chronic obstructive pulmonary disease (COPD), and respiratory infection usually accompany the symptoms of gastrointestinal discomfort (Chakradhar, 2017; Zhang et al., 2018; Wypych et al., 2019; Zhang et al., 2020). These data collectively note that there is an inevitable physiological and pathological connection between the respiratory tract and the intestine.

How it occurs is still no definite conclusion, though lots of explanations for lung-gut crosstalk have been proposed. This article aims to update and summarize the latest examples and possible mechanisms of lung-gut crosstalk in respiratory disease and IBD and discuss the role of supplement probiotics in treating diseases.

## 2 Evidence for the existence of lung-gut crosstalk

### 2.1 Aspects of pulmonary involvement in inflammatory bowel disease

IBD is an umbrella term used to describe chronic recurrent gastrointestinal inflammation, mainly including Crohn’s disease (CD) and ulcerative colitis (UC), which is a good case to study the interaction between the gut and the lung. Dirks and his colleagues reported (Dirks et al., 1989) that unexplained bronchorrhea or bronchiectasis have occurred in 6 IBD patients 3 to 13 years after their illness, it is the first time they proposed the point that IBD could involve the lungs. However, this viewpoint was questioned by some people at that time, due to only a few IBD patients displaying pulmonary complications. With the wide application of high-resolution CT scans, sensitive Pulmonary function tests, Bronchoscopy, and histological examinations, more and more IBD patients are found to have potential pulmonary dysfunction, even if they do not display obvious symptoms (Ramírez-Romero et al., 2017; Bourdillon et al., 2022; Majima et al., 2022). In the following 40 years, aspects of pulmonary involvement in IBD have been reported successively. Camus (Camus et al., 1993) found among 33 patients with ulcerative colitis (UC) or Crohn’s disease (CD), 20 cases (60%) occurred bronchiectasis, inflammatory/obliterative small airway lesions, or upper airway narrowing. Desai’s review of the literature available in 2011 showed that bronchiectasis accounted for 45% of IBD cases with large airway involvement (Desai et al., 2011). A large population-based cohort study found that compared with the non-IBD cohort, IBD patients with UC and CD had a 46% higher rate of bronchiectasis, 52% higher rate of pulmonary vasculitis and interstitial pneumonia, 35% higher risk for lung nodules, 16% higher rate of pulmonary fibrosis, and a 5.5% higher rate of asthma (Pemmasani et al., 2022). In the following content, we summarize the lung involvement in CD and UC respectively.

CD is a chronic inflammatory bowel disease of unknown etiology associated with an impaired immune response. It is characterized by patchy and transmural lesions which can affect the entire gastrointestinal tract, from the mouth to the anus (Ferré et al., 2018). Although some CD patients did not show obvious symptoms of pulmonary disease, as many as 37-55% of these patients manifested abnormalities in their chest imaging (Ateş et al., 2011; Sato et al., 2018) and pulmonary function tests (PFTs), such as the decreased of diffusing capacity for CO (DLCO) and forced expiratory volume in one second (FEV1) (Black et al., 2007). Major patterns of respiratory diseases related to CD include upper airway obstruction (Henry et al., 2001; Ahmed et al., 2005), chronic and granulomatous bronchiolitis (Camus et al., 1993; Vandenplas et al., 1998), tracheobronchitis (Asami et al., 2009), bronchiectasis (Henry et al., 2001) and asthma (Bernstein et al., 2005). By summarizing 11 lung biopsies from 11 CD patients with diffuse or local lung opacity, Casey (Casey et al., 2003) found that each patient occurred chronic bronchiolitis, non-necrotizing granulomatous inflammation, and interstitial pneumonia of varying degrees. A retrospective cohort study was conducted by using data retrieved from the Taiwan Health Insurance Research Database, patients with CD were associated with a higher subsequent risk of asthma (Peng et al., 2015). Pulmonary manifestations of CD were not only showed in humans but also in animals. Zhou et al. (2012) found that CD rats induced by 2,4,6-trinitrobenzene sulfonic acid are accompanied by symptoms of interstitial pneumonia, emphysema, and bronchiectasis.

UC is an idiopathic, chronic inflammatory disorder of the colonic mucosa (Adams et al., 2022), frequently encountered in primary care, the most common symptom is bloody diarrhea (Rubin et al., 2019). According to the literature, common pulmonary manifestations of UC include both airway disease (Camus and Colby, 2022; Majima et al., 2022) and lung disease (Massart and Hunt, 2020), which occurred in months to years after the initial presentation of UC (Majewski and Piotrowski, 2015). From 2009 to 2011, a multicenter and large-sample epidemiological survey was conducted in hospitals in Beijing, Shanghai, Henan, Jiangsu, and other places in China (Sun et al., 2011; Zhang W. et al., 2012), it was found that among UC patients, 58.6% had symptoms of shortness breath and cough; 63.3% had pulmonary function changes, which manifested as airflow limitation and decreased diffusion volume, and these symptoms were proportional to the lesion degree of UC, especially in the mild and moderate patients. Wang et al. (2012) found that the level of serum endothelin-1 (ET-1), negatively correlated with the maximal expiratory flow at 25% of forced vital capacity (FEF25), and compared with the normal group, the diffusion constant, in UC patients was significantly higher, which also suggested that the lung damage in UC patients may be related to small airway obstruction, decreased pulmonary elastic function and damaged diffusion membrane.

### 2.2 Aspects of intestinal involvement in pulmonary disease

It is noted that the “gut-lung crosstalk” is bidirectional, and respiratory diseases also involve the gut (Schuijt et al., 2016). Intestinal microbiota plays an important role in the lung-gut axis, and various lung diseases, such as COPD, cystic fibrosis, and respiratory tract infections by viruses or bacteria, can cause intestinal diseases by disturbing the composition of intestinal bacteria (Sun et al., 2020; Liu et al., 2022).

COPD is mainly caused by long-term exposure to inhalational particulate matter, such as cigarette smoke and air pollutants, also related to genetic, developmental, and social factors (Raherison and Girodet, 2009), its characteristics include persistent airflow obstruction and respiratory symptoms. A population-based cohort study found that compared with the control group, the risk of CD and UC in COPD patients increased by 2.72% and 1.83% respectively (Ekbom et al., 2008), which increased the risk of death in COPD patients (Vutcovici et al., 2016). Surprisingly, research on Quebec residents found that compared with the general population, the incidence rate of CD in COPD increased by 55% (Brassard et al., 2015). Smoking can lead to bronchitis by impairing airway epithelium and epithelial tight junction (Heijink et al., 2012), however, this cannot explain the increased prevalence of UC in COPD patients, a report showed that smoking can prevent UC in the general population (Green et al., 1996).

Asthma is a multifactorial disease characterized by an exacerbation of airway inflammation, hyperplasia of associated smooth muscle, and airway hyperresponsiveness (Cazzola et al., 2022). It occurs in early life and people are unlikely to escape from this disease in childhood (Fainardi et al., 2022). Many studies have shown that the incidence rate of asthma is closely related to the dosage of early antibiotics use. A prospective longitudinal cohort study (Strömberg Celind et al., 2018) of 4777 Swedish children found that the cumulative prevalence of asthma was 14% for those who received antibiotic treatment in the first week of life. Compared with the control group, neonatal mice, treated with antibiotics, showed significant changes in intestinal flora and exacerbation of allergic asthma, which was characterized by significantly increased of eosinophils in bronchoalveolar lavage fluid, serum ovalbumin levels, and airway hyperresponsiveness (Russell et al., 2012). In addition, some asthmatic patients are often accompanied by primary eosinophilic gastrointestinal diseases, gastroesophageal reflux disease, or severe eosinophilic esophagitis. A high level of eosinophils in both the trachea and intestine of asthmatic patients may be the key to understanding the inflammatory crosstalk of these two mucosal sites (Powell et al., 2010).

Influenza virus infection can lead to acute pneumonia and acute respiratory distress syndrome (ARDS), which were clinically defined as acute respiratory failure, and multi-organ dysfunction (Ma et al., 2020; Fanelli et al., 2022). The example of lung-gut crosstalk in chronic respiratory diseases has been introduced above. Will acute respiratory disease also affect gastrointestinal health? Studies showed that influenza patients, who have been infected with the influenza A virus (IAV) (Sencio et al., 2021) or respiratory syncytial virus (RSV) (Arias et al., 2021), often accompanied by gastroenteritis-like symptoms, such as abdominal pain, nausea, vomiting, and diarrhea, although no virus was found in the gut. Compared with normal mice, IAV-infected mice showed the symptoms of intestinal inflammation, including significant weight loss, shortened colon, damage of the intestinal mucosa, and mild diarrhea (Wang and Tian, 2015; Zhu et al., 2018). Meanwhile, interferon-gamma (IFN- γ), a common pro-inflammatory factor, was increased in IAV-treated mice intestines (Wang and Tian, 2015). Interestingly, Wang et al. found that only after the virus causes immune damage to the lungs can it cause intestinal damage (Wang et al., 2014).

## 3 Possible mechanisms of lung-gut crosstalk

**Figure 1:**
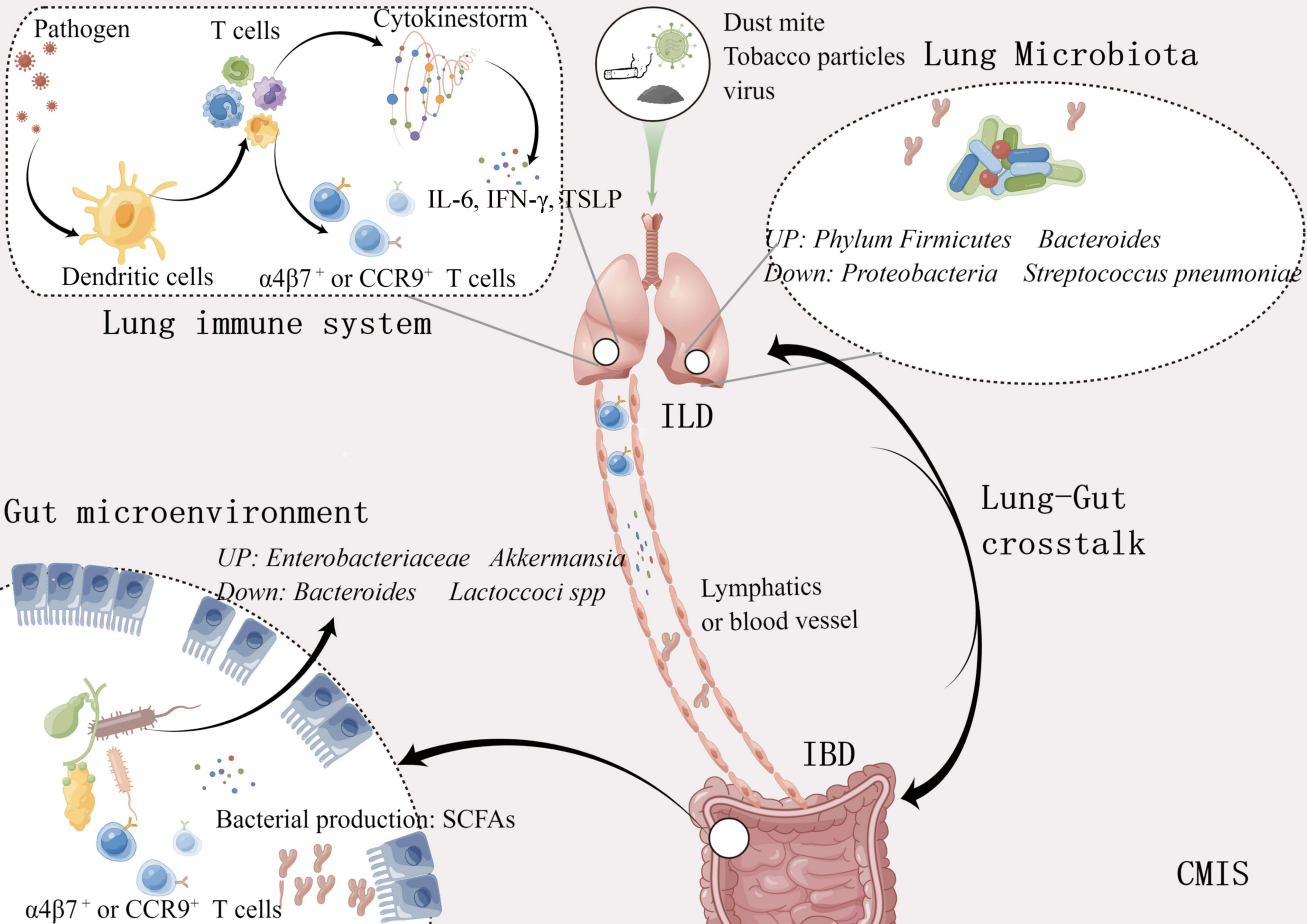
Bidirectional lung-gut crosstalk. Metabolites such as short chain fatty acids produced by intestinal bacteria move through the bloodstream, stimulating the immune response in the lungs. Different cytokines and immune cells induced by various antigens like influenza virus, and tobacco particles, also regulate the immune response of these two organs through the lymphatic vessels and blood vessels. ILD, Inflammatory lung disease; IBD, Inflammatory bowel disease; SCFAs, Short chain fatty acids; CMIS, Common mucosal immune system. This figure was made by Figdraw software.

At present, the mechanism of lung-gut crosstalk can be summarized in the following (**Figure 1**). 1) Both intestine and lung originated from the foregut region of the endoderm at the embryonic stage, with similar tissue structure and large surface area exposed to the external environment. They are the main defense organs against the invasion of foreign pathogens and have similar responses in innate immunity and adaptive immunity. 2) The respiratory system and gastrointestinal system are part of the CMIS, and lymphocytes in CMIS can crosstalk among different mucosal tissues. 3). There are lots of microbes in both the gut and lung, and various local and distant diseases are related to microbial imbalance.

### 3.1 Immune cell crosstalk between two mucosal tissues caused the immune change

It is well known that both lung and gut are mucosal tissues. More than 40 years, Mcdermott and Bienenstock (Mcdermott and Bienenstock, 1979; Mcdermott et al., 1980) proposed the concept of “common mucosal immune system” (CMIS). He speculated that CMIS, including respiratory tract, gastrointestinal tract, oral cavity and urogenital systems, is a comprehensive network composed of tissues, cells, and effector molecules, which can protect the host from infection foreign pathogens and the immune cells contained in this network can crosstalk between different mucosal tissues. More and more evidence showed that stimulating one organ of CMIS can significantly affect another mucosal tissue (Tulic and Allergy, 2016) such as intranasal vaccination can protect the genital tract from herpes simplex virus-2 (Gallichan et al., 2001), and high concentration of virus-specific immunoglobulin was found in endocervical secretions and nasal washes after HIV infection (Artenstein et al., 1997). Therefore, in CMIS “internet”, each mucosal part can effectively share information remotely.

The mucosal immune system contains about 80% of immune cells, which aggregate or transport among different mucosal tissues to maintain tissue homeostasis (Mestecky et al., 2005). Innate lymphoid cells (ILCs), a new subset of lymphocytes, are involved in innate immunity. ILCs mainly exist in the mucosal barrier and play an important role in defense against pathogens, tissue repair, and maintaining local homeostasis, therefore they are considered the first line of defense cells. During intestinal immune disorders induced by helminths or IL-25, Huang found that ILC2 could specifically migrate from the gut to the lung by the mesenteric lymphatic system and activate the inflammatory response of the lung (Huang et al., 2018). Both intestinal microorganisms and intestinal dendritic cells can improve the migration of ILC3 to the lungs by inducing the expression of CCR4 on the IL-22^+^ILC3, which eventually mediated IL-22-dependent host resistance to pneumonia in newborn mice (Gray et al., 2017). The Th17 cells, specific to both segmented filamentous bacteria (SFB) and self-antigen, could move to the respiratory tract and cause lung damage (Bradley et al., 2017). According to reported data (Bonniere et al., 1986; Fireman et al., 2000), neutrophils, eosinophils, and T cells were increased in bronchoalveolar lavage (BAL) fluid and sputum in IBD patients.

In addition, the recruitment of intestinal and respiratory effector lymphocytes was controlled by the expression of chemokine receptors or integrins. Ruane and colleagues (Ruane et al., 2013) found that pulmonary dendritic cells (DCs) can increase the expression of α4β7 and CCR9 on T cells so that these cells can migrate to the intestine with the drive of Madcam-1 and CCL25. The previous opinion was that only DCs in the gut could recruit antigen-specific T cells back to the gut. Oral probiotics can promote the proliferation of Treg cells, which can be recruited into the airway through the expression of CCR9, and where they can improve allergic airway inflammation (Navarro et al., 2011).

### 3.2 Gut microbiota are important participants in the lung-gut crosstalk

The gut is the place where microbes are majority present. In the early stage of life, with the gradual and orderly colonization of flora on the digestive tract mucosa, various microorganisms have formed a stable intestinal micro-ecosystem with a relationship of mutualism and competition (Roswall et al., 2021). However, for a long time, the lung has been considered a sterile environment, until the wide application of gene sequencing technology that the lung microbiota was gradually been found. The dynamic balance of microorganisms is essential for body health (Gubatan et al., 2021; Shan et al., 2022). In healthy individuals, the respiratory microbiota can maintain lung homeostasis by preventing the colonization of pathogenic bacteria (colonization resistance) which can be directly mediated by the competition of nutrients or the production of bacteriocins that can kill pathogenic bacteria, or indirectly mediated through the induction of local immune response, such as the production of antimicrobial peptides (Liu et al., 2020; Sequeira et al., 2020; Thibeault et al., 2021). Compared with general individuals, microbial balance is broken during respiratory disease. **Table 1** summarized the lung diseases associated with dysbiosis, including asthma, COPD, and respiratory infectious diseases.

**Table 1:** The intervention effects of the gut microbiota in respiratory diseases.

Respiratory disease	Intervention	Effects and outcomes	Underlying mechanisms	References
Asthma	Gut microbiota disrupted by antibiotics	Increased airway inflammation and Th2 responses.	Altered the Tregs subset in the colon that could, by way of the gut–lung axis, promote susceptibility to asthma.	(Yang et al., 2019)
			Enhanced Th1/Th17 adaptive immune responses in the lung.	(Russell et al., 2012)
			Altered the composition of *Micrococcaceae* and *Clostridiaceae-1*, which were potentially correlated to the infiltration of inflammatory cells.	(Russell et al., 2015)
	Probiotics	Alleviated airway inflammation.	Promoted the expansion of T-regulatory cells and IL-10.	(Durack et al., 2018)
			Increased lung CD4^+^ T cell and CD4^+^Foxp3^+^ Treg abundance while decreasing activated CD11b^+^ DC abundance.	(Raftis et al., 2018)
			Reduced the number of lung eosinophils, Th1, Th2, and Th17, and alleviate asthma symptoms	(Mazmanian et al., 2005)
COPD	Cigarette smoke	Observed gut microbiota dysregulated including enrichment of Escherichia *coli* and depletion of *Lactobacillus* spp. and ultimately caused damage to the intestinal barrier.	Activated the MAPK/ERK pathway.	(Sun et al., 2020)
			Increased the expression of mucin gene (Muc2, Muc3, and Muc4) and cytokine including CXCL2 and IL-6 while decreasing IFN-γ and TGF-β expression.	(Allais et al., 2016)
	Probiotics	Reduced the pathological manifestations of lungs and reduce inflammatory reactions.	Suppressed the activation of CS-induced NF-κB signal pathway.	(Mortaz et al., 2015)
			Increased NK cell activity and CD16 cells’ number	(Reale et al., 2012)
Respiratory infection	Influenza virus infection	Induced intestinal inflammatory injuryc	Lung-derived. CCR9+CD4+ T cells recruit into the small intestine, and increase Th7 cells by producing IFN-γ.	(Wang et al., 2014)
			Increased fecal lipocalin-2 and colonic Muc5ac levels.	(Groves et al., 2018)
	Probiotics	Decreased susceptibility to *staphylococcus aureus* pneumonia by improving bacterial burdens in the lungs, lung inflammation, and mortality	Increased levels of Th17 immune effectors such as increasing the level of IL-22 and numbers of IL-22^+^ TCR cells and neutrophils in BALF.	(Stefanie et al., 2015)
		Regulated immunity in the respiratory mucosa through the proper activation of inflammasomes.	Regulated virus-specific CD4 and CD8 T cell and antibody responses following respiratory influenza virus infection.	(Ichinohe et al., 2011)
	Gut microbiota disrupted by antibiotics	increased susceptibility to *Mycobacterium* *tuberculosis*	Reduced mincle expression on lung DCs, which reduced the activation of naïve CD4 T cells.	(Negi et al., 2019)

COPD is caused by exposure to inhalable particulate matter, especially tobacco smoke, and pollutants. It’s reported that tobacco particles could change the composition of intestinal microbes through the circulatory system, interfere with mucosal immunity, and reduce the ability of mucosa to remove toxins (Biedermann et al., 2013). Savin et al. (2018) investigated the effects of smoke on the mice gut microbiota and found that compared with the control group, smoking increased the relative abundance of *Clostridium clostridiforme* and decreased *Lactoccoci* spp. and *Ruminococcus albus*. Yu and his colleagues (Bai et al., 2022) exposed C57BL/6 mice treated with azomethane to cigarette smoke or clean air for 2 hours a day for 28 weeks. Dysregulation of gut microbiota was observed in mice, including enrichment of *Eggerthella lenta* and depletion of *Parabacteroides distasonis* and *Lactobacillus* spp. Transplanting feces from mice exposed to smoke into sterile mice, the abundance of *Escherichia coli* in the recipient mice increased, which activated the MAPK/ERK pathway, and eventually caused damage to the intestinal barrier. Furthermore, intestinal microbiota homeostasis is easily disrupted during acute exacerbations of COPD (Sun et al., 2020), the colonization number of *Helicobacter pylori* was positively correlated with the disease severity (Raftery et al., 2020) and *Haemophilus influenzae*, *Moraxella catarrhalis* and *Streptococcus pneumoniae* were found in sputum microbial of COPD patients (Bai et al., 2018). Therefore, the increase in harmful gut bacteria in COPD patients is probably the main reason for their frequent occurrence of intestinal diseases.

The diversity of pulmonary microbiota in COPD patients also changed (Mortaz et al., 2015), such as the increased abundance of *Proteobacteria* and *Actinomycetes*, and the decrease of *Phylum Firmicutes* and *Bacteroides*, which would cause pulmonary immune disorders, ultimately damage bronchial and alveolar tissues (Hufnagl et al., 2020). Oral administration of *Lactobacillus casei* can enhance the activity of immune cells in peripheral blood, and the addition of *Bifidobacterium* and *Lactobacillus rhamnosus* to mice exposed to cigarette extracts can reduce the pathological manifestations of lungs and reduce inflammatory reactions (Mortaz et al., 2015).

Mice infected with IAV presented intestinal injuries, including diarrhea, colon shortening, and disappearance of the intestinal mucosal layer, which is mainly caused by intestinal dysbiosis (Wang et al., 2014; Yildiz et al., 2018; Sencio et al., 2021). Intestinal dysbacteriosis is a common phenomenon in influenza mice, including a decrease in the ratio of *Bacteroidetes*/*Firmicutes*, and an increase in *muribaculaceae* and *porphyromonadaceae* families (Groves et al., 2018). In addition, mice infected with H3N2 or H5N1 occurred a decrease in *muribaculaceae* families (Yildiz et al., 2018; Sencio et al., 2020) and an increase in Verrucomicrobia (mainly including the *Akkerman-sia* genus) (Bartley et al., 2017; Sencio et al., 2020). However, the changes in gut microbiota after human infection with IAV are rarely reported. Gu and his colleague found that among 24 IAV patients, there is a decrease in *Actinobacteria*, *Clostridia*, and *Ruminococcaceae families* while pathogens including *Shigella* and *Escherichia species* appeared (Gu et al., 2020). In addition, the overgrowth of pathogenic bacteria such as *Escherichia coli* and *Enterococcus faecium* and the reduction of probiotics (Qin et al., 2015). *Ruminococcus* and *Bifidobacterium* were observed in patients infected with H7N9. It is necessary to investigate the gut microbiota impact of influenza virus in humans.

**Figure 2:**
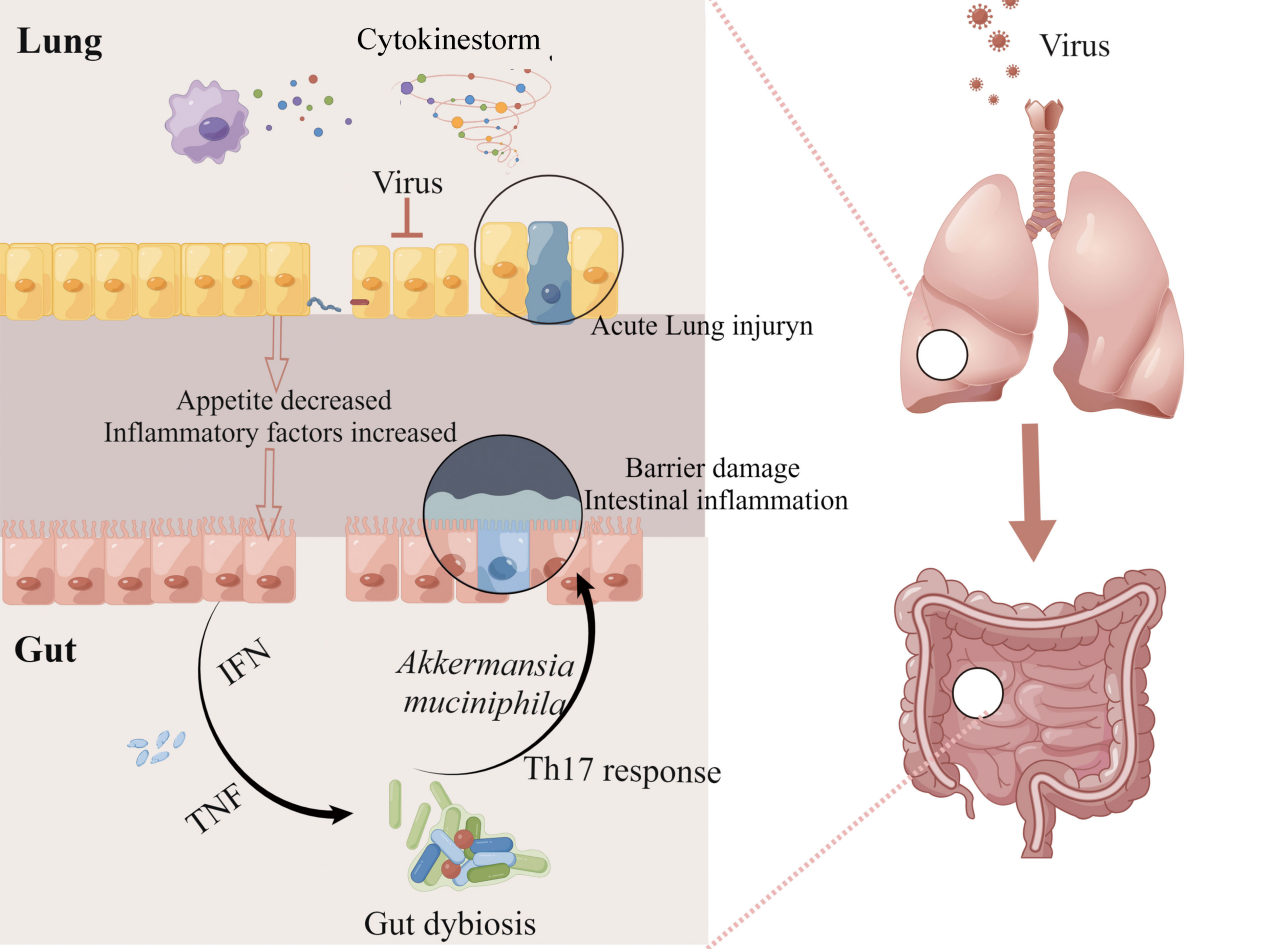
The lung-gut axis during viral respiratory infections. IAV or RAV infection can activate pulmonary immunity and cause serious lung injury. At the same time, reduced food intake and excessive secretion of cytokines such as type I and II IFNs disturbed intestinal homeostasis, and these alterations contribute to the impairment of intestinal barrier function and intestinal inflammation. This figure made by Figdraw software.

How does the influenza virus cause intestinal dysbiosis and ultimately cause intestinal damage (**Figure 2**)? There are two main reasons for intestinal dysbiosis during viral respiratory infections, one is reduced food intake and the other is the stimulation of cytokines. Type I and II IFNs, the main inflammatory factors secreted by immune cells to resist pathogens could seriously disrupt the gut microbiota during influenza (Deriu et al., 2016; Stifter et al., 2016). The deficiency of IFN-γ restored change in the relative abundance of intestinal microbiota including *Enterobacteriaceae*, *filamentous bacteria*, and *Lactobacillus genus* (Deriu et al., 2016). Mice infected with IAV or RAV showed significant weight loss mainly caused by the decrease in food intake (Monto et al., 2000; Widmer et al., 2012) and the decrease in food and calorie intake will greatly affect the diversity of gut microbiota (Wang et al., 2018). It is reported that the excessive secretion of TNF-α is one of the courses of inappetence, and TNF-α^-/-^ mice could maintain the body weight and restore the change of gut microbiota compared with normal mice infected with RSV (Groves et al., 2020). Wang and his colleagues (Wang et al., 2014) reported the link between intestinal dysbiosis and intestinal inflammation in influenza mice. They found that the increase of *Enterobacteriaceae* induced by IFN-γ produced by lung-derived CD4 T cells is the main cause of intestinal inflammation in mice infected with H1N1. As mentioned above, inflammatory cytokines can affect the diversity of gut microbiota which may cause or maintain intestinal disorders directly. *Akkermansia muciniphila* generally increased during IAV infection, with the ability to erode the colonic mucosa and contribute to the interaction between microorganisms and intestinal epithelium, which subsequently leads to the impairment of intestinal barrier function and intestinal inflammation (Groves et al., 2018). Fecal transfer experiments showed that microbiota combed from flu mice could trigger the inflammatory responses of Th17 cells in the gut of receptor mice (Wang et al., 2014). Furthermore, the clear-out of *Enterobacteriaceae* improved intestinal inflammation during H1N1 infection (Wang et al., 2014). However, the change in the intestinal microbiota was transient, and the composition would return to its original state 14 days after infection with IAV (Yildiz et al., 2018; Sencio et al., 2020).

Childhood asthma is associated with increased antibiotic exposure in the early stages of life (Ahmadizar et al., 2017; Fishman et al., 2019). Risnes et al. (2011) found that there is a positive correlation between the dose of antibiotic use in the first 6 months of life and the incidence of asthma at the age of 6. Gut microbiota plays a crucial role in the development of T helper cell 1 (Th1) and Th2 (Wu and Wu, 2012), and exposure to antimicrobial agents can delay Th1 maturation and reduce Th2 response, which is a main feature of allergy (Le and Burks, 2014; Yamamoto-Hanada et al., 2017). These reports indicate that there is a vital developmental stage in early life during which gut microbiota structure arouse the body’s systemic immune response to health.

Idiopathic pulmonary fibrosis (IPF) is a chronic, progressive, fibrotic interstitial lung disease (León-Román et al., 2022), and patients with IPF are usually accompanied by chronic intestinal inflammation and flora imbalance, such as increased *Staphylococcus*, *Streptococcus*, and *Veillonella*, and the decreased of *Bacteroides*, *Bifidobacterium adolescentis*, and *Faecalibacterium prausnitzii* (Enaud et al., 2019). There are certain bacterial species presented in IPF patience associated with immune response pathways (Huang et al., 2017; McAleer and Kolls, 2018). For example, the abundance of *Prevotella* and *Staphylococcus* in IPF patients is related to TLR9 and Th1 pathways and these changes in bacterial species are related to the circulating leukocyte phenotype, which is one of the main immune cells that cause inflammation (Huang et al., 2017). In addition, antibiotic administration increased the survival rate in IPF mice (O’Dwyer et al., 2019).

In addition, the diversity of intestinal flora in pulmonary tuberculosis patients is also reduced, and the flora producing-short chain fatty acids (SCFAs) and metabolic pathways related to SCFAs are significantly reduced (Hu et al., 2019). However, SCFAs are important for health. The study has indicated that acetate can diffuse into the blood and activate the G protein-coupled receptor (GPR) which is important for the protective effect (Dilantika et al., 2010). Butyrate provides fuel for colon epithelial cells, which is beneficial for epithelial cell metabolism, but this cell metabolism was influenced by the decrease of SCFAs.

In general, the imbalance of intestinal flora can directly or indirectly cause respiratory diseases, which can also lead to intestinal flora disorders. However, whether it is chickens laying eggs or chickens laying eggs, the above results jointly prove that the intestinal flora is essential in lung-gut crosstalk.

### 3.3 Molecules as candidates caused lung-gut crosstalk

Molecules that can cause lung-gut crosstalk were summarized in **Table 2**, Lipopolysaccharide (LPS), the main component of the cell wall of gram-negative bacteria (Farhana and Khan, 2023), can stimulate toll-like receptor (TLR) 4 and activate the innate immune response of the host (Neyen and Bruno, 2016; Nighot et al., 2017). Appropriate stimulation of LPS in the intestine is beneficial to the immune response of the lungs. The resistance of mice with exhausted intestinal flora to pneumonia induced by the influenza virus or *E. col*i- is greatly reduced, but it was significantly improved when LPS is administered simultaneously with antibiotics (Shi et al., 2021). Similarly, Th2 response was decreased after being administered with LPS in asthma mice which suggested that LPS has a preventive effect on asthma (Qian et al., 2018). However, intratracheal instillation of LPS could significantly increase the number of bacteria in the cecum within 24 hours (Tang et al., 2021) which may break the intestinal immune homeostasis. After the invasion of pathogens into lung tissue, lymphocytes in the lung secrete amounts of cytokine that induce cytokine storm. Thymic stromal lymphopoietin (TSLP), is critical to induce the activation of both innate and adaptive immune responses in asthma. It can activate the STAT5 signaling pathway, and mediate eosinophils recruitment and type 2 cytokines secretion by ILC2 or Th2 cells (Lin et al., 2016; Salvati et al., 2021). IL-6, one of the main inflammatory cytokines during infectious respiratory diseases, is the main activating factor of Th17 cells subset which can drive Th17 inflammatory response in multiple organs (Vargas-Rojas et al., 2011; Lin et al., 2016) and regulate the number and phenotype of microbe-responsive regulatory T cells in the gut (Yan et al., 2021). Other candidates mainly include growth factors and cytokines, such as vascular endothelial growth factor (VEGF) (Larsson-Callerfelt et al., 2020), Proteases (Reed and Kita, 2004; Chen et al., 2022), Interferon (IFN) (Erttmann et al., 2022), SCFAs(Theiler et al., 2019; Richards et al., 2020), Tumor Necrosis Factor (TNF) (Rutting et al., 2019), Tobacco particles (Baskara et al., 2020; Danov et al., 2020) and so on, which are closely related to IBD and respiratory diseases.

**Table 2:** The intervention effects of molecules in lung-gut crosstalk.

Molecules	Underlying mechanisms	References
VEGF	VEGF is a mediator of IBD by promoting intestinal angiogenesis and inflammation and it also can promote pulmonary angiogenesis, leukocyte extravasation, inflammation, and decreased endothelial barrier function in the lungs.	(Laddha and kulkarni, 2019); Larsson-Callerfelt et al., 2020; Li et al., 2020
IL-6	IL-6 is major triggers in the development of Th17 subset of T cells which may drive cross-organ Th17 polarized inflammation.	(Vargas-Rojas et al., 2011; Lin et al., 2016; Yan et al., 2021)
TSLP	The breakdown of TSLP results initiation of Th2 cytokines, IgE production and Th2-mediated allergic diseases.	(Takai, 2012; Salvati et al., 2021)
Proteases	Increased of protease activity leading to a defective epithelial barrier and excessive inflammation.	(Reed and Kita, 2004; McKelvey et al., 2021; Chen et al., 2022)
LPS	Stimulation of LPS is beneficial to the immune response of the lungs and increases the number of bacteria in the cecum.	(Tang et al., 2021; Neyen et al., 2016; Nighot et al., 2017)
IFN	Gut microbiota can prime systemic antiviral immunity *via* the cGAS-STING-IFN-I axis.	(Erttmann et al., 2022)
Dermatophagoides pteronyssinus (Der p1)	Der p1 is the major respiratory allergen from house dust mite and has detrimental effect in the healthy gut and lungs.	(Tulic et al., 2016)
SCFAs	Oral administration of SCFAs can reduce the migration of eosinophils and restore the barrier function of damaged airway epithelium by increasing the expression of ZO-1 dense contact protein.	(Theiler et al., 2019; Richards et al., 2020)
Tobacco particals	Smoke can damage airway epithelia, affect maturation of lung DCs, lymphocyte homing and trafficking, and selectively inhibit bacterial growth	(Keely and Hansbro, 2014; Baskara et al., 2020; Danov et al., 2020)

## 4 Treatment of respiratory diseases based on intestinal microorganisms

To sum up, there is a close relationship between gut microbiota and host health, therefore, studies on regulating microbial diversity to treat diseases, especially respiratory diseases are increasing. Presently, the common methods used to regulate microbiota include a high-fiber diet and supplement of probiotics (especially *lactic acid bacteria* and *bifidobacteria*).

Probiotics are living microorganisms, which play a critical role in activating host immunity and resisting pathogens. In addition to gastrointestinal diseases, probiotics also exhibit good therapeutic effects in respiratory diseases (Debnath et al., 2022; Huang et al., 2022). It has been reported that oral administration of *Bifidobacterium brevis* (Sagar et al., 2014), *Lactobacillus rhamnosus* (Spacova et al., 2020), and *Bifidobacterium lactis* (Panebianco et al., 2019) can induce antigen-specific reactions, which contribute to inhibit allergic reactions. Similarly, *Enterococcus faecalis* FK-23 can inhibit allergic airway inflammation by reducing Th 17 responses (Zhang B. et al., 2012). In addition, tobacco particles and tobacco toxins could affect mucosal immunity and reduce the ability of mucosa to remove toxins by changing the structure of the intestinal microbial community (Allais et al., 2016). Administer *Lactobacillus casei*, *bifidobacterial*, and *Lactobacillus rhamnosus* to mice exposed to cigarettes could enhance the immune cells’ activity in peripheral blood and reduce the pathological manifestations and the inflammatory response of lungs (Mortaz et al., 2015). In a randomized clinical trial, infant formula rich in galactooligosaccharides/polydextrose increases the colonization of protective bacteria in the gut, including *Bifidobacteria* and *Clostridium*, thus protecting infants born to atopic parents from respiratory tract infections (Ranucci et al., 2018).

Diet is one of the main reasons that directly cause changes in gut microbiota (Moszak et al., 2020). Wang (Wang et al., 2020) found that high-fat diet (HFD) reduced the abundance of *Lactobacillus*, *Bifidobacterium*, *Akkermansia*, *Faecalibaculum*, and *Blautia* and the predicted metabolic pathways indicated that HFD increased the risks of intestinal pathogens colonization and inflammation. Therefore, adjusting diet is an effective method to adjust the structure of intestinal bacteria in addition to supplementing probiotics. It has been noted that carbohydrates play a beneficial role in shaping intestinal probiotics. Complex carbohydrates, specifically these available for intestinal probiotics, such as fructooligosaccharides and galactooligosaccharides have a crucial role in the growth of *Bacteroides*, *Bifidobacterium*, and *Lactobacillus*, which is essential in the production of SCFs (Lamuel-Raventos and Onge, 2017; Nicolucci et al., 2017). SCFA produced by complex carbohydrates can reduce asthma responses (Trompette et al., 2014) and prevent the progression of allergic respiratory inflammation (Trompette et al., 2014; Tian et al., 2019; Yip et al., 2021) by impairing pathogenic type 2 immune responses and inhibiting the activity of histone deacetylase (Tedelind et al., 2007). An experimental study proved that the consumption of fiber (especially the soluble part) at the optimal amount (about 30 g/d) is positively related to the amount of butyrate produced by a variety of bacteria including *Faecalibacterium prausnitzii*, *Clostridium*, *Butyrivibrio* and *Eubacterium* (Scott et al., 2008). Increasing the intake of vegetables and fruits rich in soluble fiber can reduce the risk of developing COPD and improve symptoms of COPD such as breathlessness (Tabak et al., 2001; Watson et al., 2002).

In all, both supplementing probiotics and adjusting diet are aimed at shaping a healthy intestinal flora for the treatment of respiratory diseases. Compared to traditional drugs, this treatment is safer, but more prone to the treatment of chronic diseases.

## 5 Conclusions and outlook

Both the gut and lung originating from the foregut region of the endoderm have similar immunological characteristics, which led to these two mucosal organs having similar barrier functions, such as controlling the transport of gas or nutrients and preventing pathogens from entering the host. Similar functions make two tissues respond similarly to pathogenic bacteria, which may be a reasonable explanation for the occurrence of lung-gut crosstalk.

Lung-gut crosstalk was first discovered 40 years ago. Lots of explanations for lung-gut crosstalk have been proposed, but most of them lack strong evidence. Currently, the mechanism of the lung-gut axis most focuses on the intestinal and pulmonary microbiota and the common mucosal immune system. There are many studies focusing on intestinal dysbiosis in respiratory diseases, however, the question of whether this imbalance is the cause or the consequence of respiratory diseases is not been resolved. In addition, research on inter-communication between mucosal immune organs also needs to be strengthened. Clarifying the mechanism of lung-gut crosstalk is very important for us to explore the etiology of complex mucosal diseases such as IBD, COPD, and allergy. There are many ways to regulate microorganisms to treat respiratory diseases, in addition to the above-mentioned methods such as prebiotics supplements and diet adjustment, fecal microbiota transplantation (FMT) is also a good way. Meanwhile, environmental factors should not be ignored, such as antigens found in the dust we are constantly exposed to every day, which is in its infancy and has great prospects in finding better treatment methods for mucositis diseases in the future.

## Author contributions

BD, YF, and YH conceptualized and wrote the manuscript; QS, YY, and RR revised the article critically for important intellectual content. JX collected references for this manuscript and played a guiding role in the drawing of figures. All authors contributed to the article and approved the submitted version.
